# Processing and Analysis of Multichannel Extracellular Neuronal Signals: State-of-the-Art and Challenges

**DOI:** 10.3389/fnins.2016.00248

**Published:** 2016-06-02

**Authors:** Mufti Mahmud, Stefano Vassanelli

**Affiliations:** NeuroChip Laboratory, Department of Biomedical Sciences, University of PadovaPadova, Italy

**Keywords:** neuroengineering, brain-machine interface, neuronal probes, neuronal signal, neuronal signal processing and analysis, neuronal activity, neuronal spikes, local field potentials

## Abstract

In recent years multichannel neuronal signal acquisition systems have allowed scientists to focus on research questions which were otherwise impossible. They act as a powerful means to study brain (dys)functions in *in-vivo* and in *in-vitro* animal models. Typically, each session of electrophysiological experiments with multichannel data acquisition systems generate large amount of raw data. For example, a 128 channel signal acquisition system with 16 bits A/D conversion and 20 kHz sampling rate will generate approximately 17 GB data per hour (uncompressed). This poses an important and challenging problem of inferring conclusions from the large amounts of acquired data. Thus, automated signal processing and analysis tools are becoming a key component in neuroscience research, facilitating extraction of relevant information from neuronal recordings in a reasonable time. The purpose of this review is to introduce the reader to the current state-of-the-art of open-source packages for (semi)automated processing and analysis of multichannel extracellular neuronal signals (i.e., neuronal spikes, local field potentials, electroencephalogram, etc.), and the existing Neuroinformatics infrastructure for tool and data sharing. The review is concluded by pinpointing some major challenges that are being faced, which include the development of novel benchmarking techniques, cloud-based distributed processing and analysis tools, as well as defining novel means to share and standardize data.

## 1. Introduction

The open question of structure-function relationship has attracted lot of interests in Systems Neuroscience. Recent works on anatomical substructures of the brain (Briggman and Denk, 2006; Mikula et al., 2012) promise to improve our understanding of neuronal networks physiology and drive the development of novel applications of neurotechnology by interpreting the activities of large neuronal ensembles via extracellular methods (Buzsaki, 2004; Nicolelis and Lebedev, 2009).

On the other hand, neuronal signals recorded by means of neuronal probes require rigorous (pre)processing and analysis. In terms of technological advancement, the extracellular interfacing of neurons with artificial chip-based devices has taken a considerable leap forward, even in comparison with very popular patch-clamp, EEG, and fMRI techniques (Vassanelli, 2011; Spira and Hai, 2013). In the last two decades, such advances have allowed neuroscientists to record neural activity simultaneously from many neurons with up to thousands of recording sites in a single neuronal probe and at a temporal resolution from a few up to hundreds of kilo Hertz (kHz) (Buzsaki, 2004; Schröder et al., 2015).

**Figure 1:**
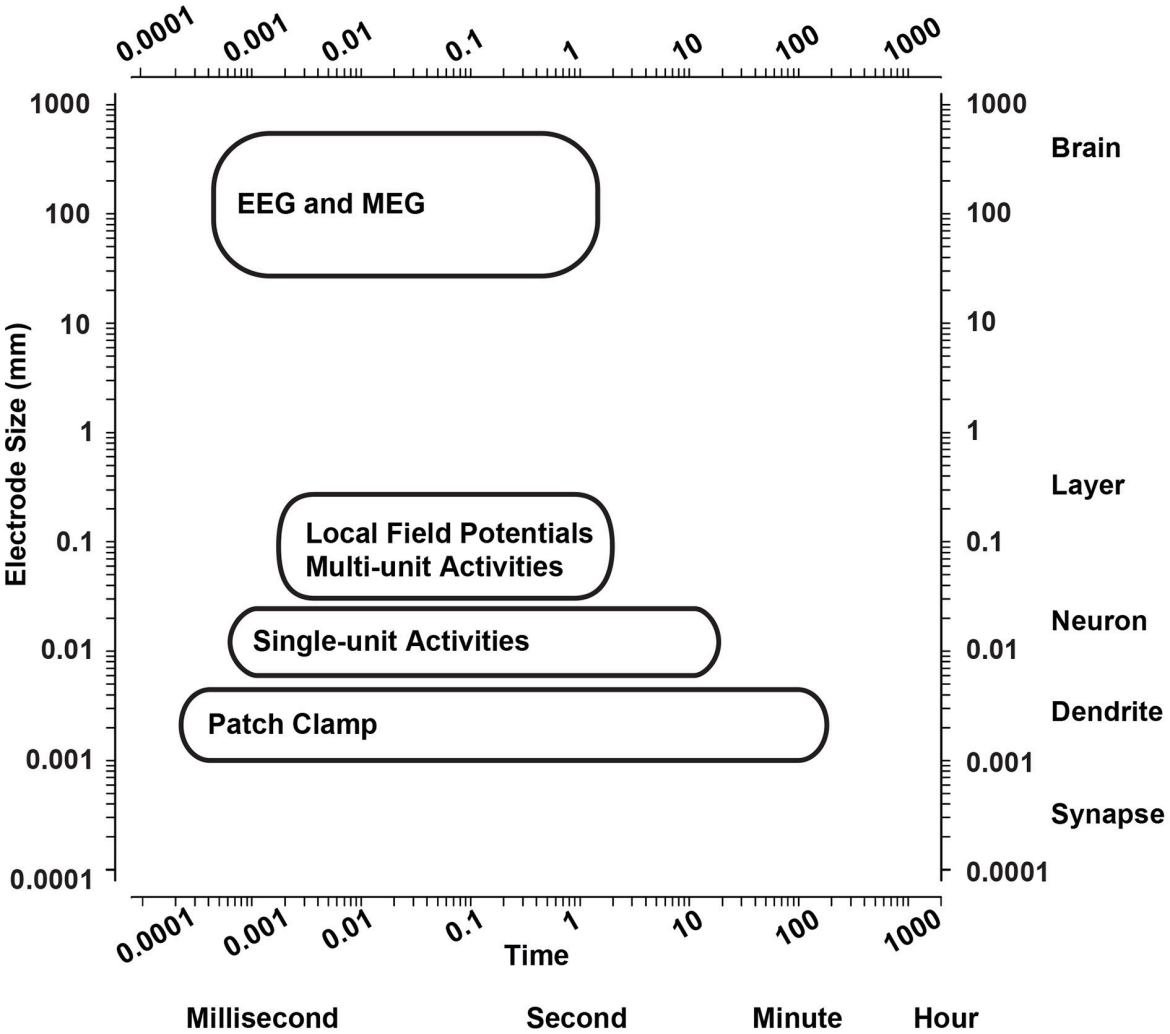
**Spatiotemporal range of neurophysiological signal acquisition techniques**. Spatiotemporal range of the main techniques to measure neurophysiological signals from the brain. EEG, electroencephalography; MEG, magnetoencephalography.

The wide variety of electrode size and dimensions allow different types of neuronal signals to be recorded from the extracellular space. Single-unit activities (action potentials) from single neurons can be sensed by small electrodes in their close proximity (Buzsaki et al., 2012). They also pick multi-unit activities from several simultaneously active neurons nearby to the electrode (Einevoll et al., 2012). With increasing electrode dimensions, local field potentials (LFPs) are sensed from neighboring neuronal populations as synchronous net activity of several hundreds to thousands neurons (Tsytsarev et al., 2006; Vassanelli, 2011, 2014; Vassanelli et al., 2012; Khodagholy et al., 2015). Therefore, the neurophysiological signals from different brain structures can be measured using a wide range of techniques based on the dimensions of the electrodes (see Figure 1; Sejnowski et al., 2014).

Also, the massive growth in the field of brain imaging techniques allowed scientists to image brain activities at very different scales, from imaging single ion-channels to the whole brain (for a review, see Freeman, 2015).

Recently developed neural probes allowed neuroscientists to investigate neural processing by monitoring groups of neurons and their activation patterns at unprecedented resolution (Brown et al., 2004; Giocomo, 2015), thus also contributing to bridge the gap between neuronal network activity and behavior (Berenyi et al., 2014). In addition, they provided deep insights on the pathological basis of brain disorders (Friston et al., 2015). As a drawback, investigation of brain function and pathology can require massive data mining. For example, in an hour, a 128 channel signal acquisition system with 16 bits A/D conversion and 20 kHz sampling rate will generate approximately 17 GB uncompressed data (Mahmud et al., 2014). Inferring meaningful conclusions from this massive amount of data is pivotal to the neuroscience and neuroengineering community (Mahmud et al., 2010a, 2012a) and tools for analysis of such multichannel extracellular recordings that support a rapid and accurate data interpretation are still missing (Stevenson and Kording, 2011). Though computing power increased and costs decreased, yet, processing and analysis of signals remained labor-intensive. This poses a huge challenge to the computational neuroscientists: to develop tools to analyze such complex data that are optimized for both memory management and processing times (Stevenson and Kording, 2011).

Over the years, to make data handling and analysis fast, interactive and user friendly, several software tools have been developed by individual laboratories, e.g., Mahmud et al. (2012a), but only a negligible number of them have been released to the community. In practice, large number of analysis scripts are kept private, leading to a situation where analysis transparency is reduced and reproducibility of analysis results is hampered (Schofield et al., 2009).

It has also been argued that the acquired data, despite being in digitized form, have been only minimally made publicly available for other scientists to explore and validate (Van Horn and Ball, 2008). To overcome this, in recent years, the community sees a growing need to have standardized and publicly available tools (Gardner et al., 2008; Akil et al., 2011) as well as experimental data repositories (Ascoli, 2006a; De Schutter, 2010). To this aim, a paradigm shift has been initiated by a set of laboratories to share their analysis tools through open-source licenses fostering standardization (Ince et al., 2010). Given the circumstances, distributed and cloud-based computing solutions have become an obvious and valuable option (Mahmud et al., 2014).

This review will introduce the readers to the available major open-source academic toolboxes for processing and analysis of neurophysiological signals acquired by means of multichannel probes, and the available infrastructure for sharing such tools and the experimental data. Also, some of the challenges and bottlenecks the community is currently facing will be identified and highlighted, and development perspectives which, in our opinion, will facilitate result reproducibility, flexibility, and standardization will be provided.

## 2. State-of-the-art

The state-of-the-art for processing and analysis of neurophysiological signals can be categorized based on signal types, i.e., electroencephalography or magnetoencephalography (local) field potentials, and spikes. Though the majority of the toolboxes specialize to process and analyze one specific type of signal, there exist a few which provide rather comprehensive methods covering two or more signal types. Therefore, based on the signal types we categorized the toolboxes into three broad categories:

- Toolboxes for Electroencephalography (EEG) analysis;
- Toolboxes for spike trains and field potentials analysis;
- Toolboxes for spike sorting.

Most of the tools were developed mainly in Matlab (Mathworks Inc., Natick, USA; www.mathworks.com) and python (www.python.org) programming languages due to their diffused usage in the neuroscience community. Other programming languages such as C, C++, R, Delphi7, and Java were also used in partial coding of some packages.

### 2.1. Toolboxes for electroencephalography (EEG) analysis

In the last decade, various techniques have been developed and applied to EEG data analysis and focused reviews on specific techniques have been reported (Pascual-Marqui et al., 2002; Stam, 2005; Hallez et al., 2007; Grech et al., 2008; Lenkov et al., 2013; de Cheveigné and Parra, 2014). Table 1 summarizes some of the popular open-source EEG analysis tools with their representative features which are enlisted below.

**Table 1:** **Popular EEG processing and analysis toolboxes with their representative features**.

**Toolbox**	**Features**								
	**Lang**	**PF**	**GUI DV**	**DIE**	**AR**	**E/C L**	**SM**	**Representative method(s)**	**Representative measure/analysis**
EEGLAB	Matlab	LUMW	Yes	Yes	Yes	Yes	Yes	ICA (Comon, 1994)	1. ERSP; 2. ITC; 3. ERCC
FieldTrip	Matlab	OSML	Yes	Yes	Yes	Yes	Yes	1. Fourier / WT (Perrier et al., 1995); 2. MTM (Percival and Walden, 1993); 3. LCMVB (Veen and Buckley, 1988); 4. MUSIC (Schmidt, 1986)	1. (N)PSA; 2. BMCA
ERPWAVELAB	Matlab	LUMW	Yes	Yes	Yes	Yes	Yes	1. PARAFAC (Harshman, 1970); 2. TUCKER (Tucker, 1966)	1. ESP; 2. ITPC; 3. ITLC
eConnectome	Matlab	OSML	Yes	Yes	No	Yes	Yes	1. GC (Granger, 1969); 2. (Adaptive) DTF (Kaminski and Blinowska, 1991; Wilke et al., 2008); 3. PDC (Baccala and Sameshima, 2001); 4. CCD (Dale and Sereno, 1993)	1. EERP; 2. ERS/D; 3. FC
pyMVPA	Python	LMW	Yes	Yes	No	No	No	1. LSVM (Vapnik, 1995); 2. SMLR (Krishnapuram et al., 2005); 3. GPRLK (Rasmussen and Williams, 2006)	1. MIRELIEF; 2. ANOVA
SCoT	Python	NS	No	No	No	No	Yes	1. MVARICA (Koles et al., 1990); 2. CSPVARICA	1. CSD; 2. (FF/G)P(D)C; 3. (D/FF/G)DTF; 4. F/EC
EMDLAB	Matlab	OSML	Yes	No	No	No	No	(E/wS/M)EMD (Rehman and Mandic, 2010; Rutkowski et al., 2010; Zeiler et al., 2012)	1. IMF; 2. ERM
PREP	Matlab	OSML	Yes	Yes	Yes	No	No	1. MTM (Mitra and Pesaran, 1999); 2. ICA; 3. PCA; 4. LDA & HDCA (Marathe et al., 2014)	1. MAC; 2. RWD
EEGVIS	Matlab	OSML	Yes	No	No	No	No	Provides rich visualization of multichannel EEG	−

*Lang, Language; PF, Platform; GUI DV, GUI and Data Visualization; DIE, Data Import/Export; AR, Artifact Removal; E/C L, Event/Channel Localization; SM, Source Modeling; L, Linux; U, Unix; M, Mac; W, Windows; OSML, Operating System supported by Matlab; NS, Not Specified; ICA, Independent Component Analysis; WT, Wavelet Transform; MTM, MultiTaper Methods; PARAFAC, PARAllel FACtor analysis; LCMVB, Linear Constrained Minimum Variance Beamformer; LSVM, Linear Support Vector Machine; SMLR, Sparse Multinomial Logistic Regression; GPRLK, Gaussian Process Regression with Linear Kernel; GC, Granger Causality; DTF, Directed Transfer Function; PDC, Partial Directed Coherence; CCD, Cortical Current Density; MVARICA, Multi Vector AutoRegrassive Independent Component Analysis; CSPVARICA, Common Spatial Patterns Vector AutoRegrassive Independent Component Analysis; (E/wS/M)EMD, (Ensemble/weighted Sliding/Multivariate) Empirical Mode Decomposition; ERSP, Event-Related Spectral Perturbation; ITC, Inter-Trial Coherence; ERCC, Event-Related Cross-Coherence; (N)PSA, (Non)Parametric Spectral Analysis; BMCA, Bivariate and Multivariate Connectivity Analysis; ESP, Evoked Spectral Perturbation; ITP/LC, Inter-Trial Phase/Linear Coherence; MIRELIEF, Multivariate Iterative RELIEF; EERP, Extraction of Event-Related Potentials; ERS/D, Event-Related Synchronization/Desynchronization; F/EC, Functional/Effective Connectivity; CSD, Cross Spectral Density; (FF/G)P(D)C, (Full Frequency/Generalized) Partial (Directed) Coherence; (D/FF/G)DTF, (Direct/Full Frequency/Generalized) Directed Transfer Function; IMF/ERM, Visualization of (Intrinsic Mode Functions/Event-Related Modes); MAC, Maximum Absolute Correlation; RWD, Robust Window Deviation*.

#### 2.1.1. EEGLAB

“EEGLAB” is a Matlab based EEG signal processing environment with time-frequency and ICA methods (Delorme and Makeig, 2004). It allows the user to: plot channel spectra and maps, remove artifacts, extract signal epochs, average data, select and compare multiple data, plot event related potential (ERP) images, decompose data using ICA and time/frequency methods, and estimate source locations. In addition, it also allows handling data from multiple subjects and perform statistical analysis on them. It can be obtained from http://sccn.ucsd.edu/eeglab/.

#### 2.1.2. ERPWAVELAB

“ERPWAVELAB” is another Matlab based EEG processing toolbox (Morup et al., 2007) which depends on EEGLAB for certain functionalities. It is capable of multi-channel time-frequency analysis of ERP of EEG and MEG data. Provides data decomposition using multiway (tensor) factorization. The features include: various visualizations and maps, artifact rejection in the time-frequency domain, clustering dendrogram, statistical analysis across different groups and subjects, cross coherence analysis, etc. It can be obtained from www.erpwavelab.org.

#### 2.1.3. pyMVPA

“pyMVPA” is a multivariate pattern analysis package developed in Python and aims to facilitate statistical learning analyses of large datasets (Hanke et al., 2009). It offers data handling and an extensible framework for multivariate statistical analyses such as, classification, regression, and feature selection. It can be downloaded from www.pymvpa.org/.

#### 2.1.4. eConnectome

“eConnectome” is a Matlab based software with interactive graphical interfaces for EEG/ECoG/MEG preprocessing, source estimation, connectivity analysis and visualization where the connectivity from EEG/ECoG/MEG can be mapped over sensor and source domains (He et al., 2011). It can be obtained from http://econnectome.umn.edu/.

#### 2.1.5. FieldTrip

“FieldTrip” is a Matlab based toolbox developed for the analysis of MEG, EEG, and other noninvasively recorded electrophysiological data (Oostenveld et al., 2011). Capable of handling data directly from many proprietary formats (e.g., BrainProducts/BrainVision, NeuroScan, Electrical Geodesics Inc., BCI2000, Micromed, Nexstim, European data format, Generic standard formats, etc.), it provides the user to perform time-frequency analysis using multitapers, source reconstruction using dipoles, distributed sources and beamformers, connectivity analysis, and nonparametric statistical permutation tests at the channel and source level. It can be obtained from www.fieldtriptoolbox.org.

#### 2.1.6. EEGVIS

“EEGVIS” is a Matlab based toolbox that allows users to explore multichannel EEG and other large array-based data sets using multiscale drill-down techniques (Robbins, 2012). Available at http://visual.cs.utsa.edu/research/projects/eegvis, and useable as a plugin to “EEGLAB.”

#### 2.1.7. SCoT

“SCoT” is a toolbox written in Python for connectivity analysis on EEG/MEG sources. It performs blind source separation, connectivity estimation, resampling statistics, and visualization (Billinger et al., 2014). It works with both multi-trial and single trial data. The source code can be downloaded from https://github.com/SCoT-dev/SCoT.

#### 2.1.8. EMDLAB

“EMDLAB” is developed in Matlab as a plugin to the EEGLAB to perform various empirical mode decomposition (EMD), e.g., plain EMD, ensemble EMD, weighted sliding EMD, and multivariate EMD (MEMD) on EEG data (Al-Subari et al., 2015). It can be obtained from http://sccn.ucsd.edu/eeglab/plugins/EMDLAB_Plugin.zip.

#### 2.1.9. PREP

“PREP” is for early-stage EEG processing which is a Matlab based preprocessing pipeline that aims in cleaning (e.g., line noise removal, fixing drifting problem, interpolating corrupt channels, etc.) the EEG signals (Bigdely-Shamlo et al., 2015). The library is available at http://eegstudy.org/prepcode.

### 2.2. Toolboxes for spike trains and field potentials analysis

With the increasing capabilities to record simultaneously from a growing number of neurons, computational neuroscientists developed automated toolboxes addressing the required processing and analyses. We touch upon few of the publicly available ones below. Table 2 summarizes the packages we discuss below with their representative features.

**Table 2:** **Popular spike trains and field potentials processing and analysis toolboxes with their representative features**.

**Toolbox**	**Features**						
	**Lang**	**PF**	**GUI DV**	**DIE**	**AR**	**PDP**	**Representative method(s)/measures/analyses**
DATA-MEAns	Delphi7	W	Yes	No	No	No	1. PoSTH; 2. PEH; 3. AC; 4. CC; 5. FF; 6. COH; 7. ES (Quian Quiroga et al., 2002); 8. NNC (Cover and Hart, 1967); 9. KMC (MacQueen, 1967)
MeaBench	C++ / Matlab	L	Yes	No	Yes	No	1. Spike detection; 2. Spike validation; 3. Burst detection
KNSNDM	C++	LMW	Yes	Yes	Yes	No	1. AC; 2. CC; 3. KlustaKwik^2.3.2^; 4. CEM (Celeux and Govaert, 1992)
BSMART	Matlab / C	OSML	Yes	Yes	No	No	1. AMAR; 2. (A)B/MAR; 3. FFT; 4. GC (Granger, 1969); 5. COH; 6. CN; 7. GCN
FIND	Matlab	OSML	Yes	Yes	No	No	1. CV; 2. PPM; 3. PWCC; 4. ASGF; 5. RLDE (Nawrot et al., 2003); 6. Spike detection
STAToolkit	Matlab / C	LMW	Yes	No	No	Yes	1. DM (Strong et al., 1998); 2. MSM (Victor and Purpura, 1997); 3. BLM (Victor, 2002); 4. AD (Treves and Panzeri, 1995); 5. JD (Thomson and Chave, 1991); 6. DMB (Ma, 1981); 7. BUB (Paninski, 2003); 8. CA (Chao and Shen, 2003); 9. BDP (Wolpert and Wolf, 1995)
PANDORA	Matlab	LMW	No	Yes	No	EMP	1. RDBCDS; 2. SSC; 3. KLDM (Kullback and Leibler, 1951); 4. RAD (Johnson and Sinanovic, 2001)
sigTOOL	Matlab	OSML	Yes	Yes	No	No	1. AC; 2. CC; 3. COH; 4. PSA; 5. ICA; 6. PEH
ibTB	Matlab	LMW	No	No	No	No	1. DM; 2. QE (Strong et al., 1998); 3. PTBC (Panzeri and Treves, 1996); 4. SHP (Montemurro et al., 2007); 5. BBC (Optican et al., 1991); 6. GM (Misra et al., 2005)
Chronux	Matlab	LMW	Yes	No	No	No	1. HCM (Fee et al., 1996); 2. LOWESS (Cleveland, 1979); 3. LOCFIT (Loader, 1999); 4. MTM; 5. COH; 6. SFC
SPKTool	Matlab	OSML	Yes	Yes	No	No	1. (NL)ESD (Mukhopadhyay and Ray, 1998); 2. PCA; 3. EMGMM (Duda et al., 2000)
nSTAT	Matlab	OSML	No	No	No	No	1. PPGLM (Paninski et al., 2007); 2. GLM-PSTH; 3. (A/B)IC; 4. SSGLM; 5. KF; 6. MTM; 7. STG
SigMate	Matlab	OSML	Yes	Yes	Yes	No	1. FO; 2. LC (Mahmud et al., 2016); 2. CLAOD (Mahmud et al., 2010b); 3. CSD (Mahmud et al., 2011); 4. SLFPC (Mahmud et al., 2012c)
MVGC	Matlab	OSML	No	No	No	No	1. OLS; 2. LWRA (Levinson, 1946); 3. VARMLE; 4. CPSD; 5. MTM; 6. FFT; 7. UGC
QSpike Tools	Matlab	ML	No	No	Yes	EMP	1. Spike detection; 2. Spike validation; 3. PSTH; 4. PEH; 5. Burst detection and validation; 6. Wave_Clus^Section 2.3.1^

*Lang, Language; PF, Platform; GUI DV, GUI and Data Visualization; DIE, Data Import/Export; AR, Artifact Removal; PDP, Parallel Data Processing; KNSNDM, Klusters, NeuroScope, NDManager; L, Linux; U, Unix; M, Mac; W, Windows; OSML, Operating System supported by Matlab; PoSTH, Post-Stimulus Time Histogram; PSTH, Peri-Stimulus Time Histogram; PEH, Peri-Event Histogram; AC, AutoCorrelation; (PW)CC, (Pair Wise) Cross-Correlation; FF, Fano Factor; COH, (Cross) COHerence; ES, Event Synchrony; NNC, Nearest Neighbor Clustering; KMC, K-Means Clustering; CEM, Classification Expectation Maximization; (A)B/MAR, (Adaptive) Bi/Multi variate AutoRegrassive model; FFT, Fast Fourier Transform; GC, Granger Causality; CN, Coherent Network; GCN, GC Network; PPM, Point Process Model; ASGF, Asymmetric SavitzkyGolay Filter; RLDE, Response Latency Differences Estimation; DM, Direct Method; MSM, Metric Space Method; BLM, BinLess Method; AD, Asymptotically Debiased; JD, Jackknife Debiased; DMB, Debiased Ma Bound; BUB, Best Upper Bound; CA, Coverage-Adjusted; BDP, Bayesian with Dirichlet Prior; EMP, EMbarrassingly Parallel; RDBCDS, Rational DataBase Creation from DataSet; SSC, Spike Shape Characteristics; KLDM, Kullback-Leibler Divergence Measure; RAD, Resistor-Average Distance; PSA, Power Spectral Analysis; QE, Quadratic Extrapolation; PTBC, Panzeri and Treves Bias Correction; SHP, Shuffling Procedure; BBC, Bootstrap Bias Correction; GM, Gaussian Method; HCM, Hierarchical Clustering Method; LOWESS, Locally Weighted Sum of Squares; SFC, Spike Field Coherence; (NL)ESD, (NonLinear) Signal Energy for Spike Detection; EMGMM, Expectation Maximization on Gaussian Mixed Model; PPGLM, Point Process Generalized Linear Model; (A/B)IC, (Akaike's/Bayesian) Information Criteria; SSGLM, State-Space Generalized Linear Model; KF, Kalman Filtering; STG, Spectrogram; FO, File Operations (file splitting, concatenation, column rearranging); LC, Latency Calculation; CLAOD, Cortical Layer Activation Order Detection; CSD, Current Source Density; SLFPC, Single LFP Classification; OLS, Ordinary Least Squares; LWRA, LWR Algorithm; VARMLE, VAR Maximum Likelihood Estimator; CPSD, Cross-Power Spectral Density; UGC, Unconditional GC*.

#### 2.2.1. DATA-MEAns

“DATA-MEAns” is a toolbox developed in Borland Delphi 7 (Embarcadero Technologies Inc., Austin, USA) and Matlab (Bonomini et al., 2005). It provides data visualization, basic analysis (i.e., autocorrelations, perievent histograms, rate curves, PSTHs, ISIs, etc.), and nearest neighbor or k-means clustering. Available at http://cortivis.umh.es/.

#### 2.2.2. MeaBench

“MeaBench” is a toolbox written mainly in C++ with certain parts written in Perl^1^ and Matlab. It is intended for data acquisition and online analysis of commercial multielectrode array recordings from Multichannel Systems GmbH (Reutlingen, Germany) (Wagenaar et al., 2005). It allows real-time data visualization, line and stimulus artifact suppression, spike and burst detection and validation. Available at www.danielwagenaar.net/res/software/meabench/.

#### 2.2.3. Klusters, NeuroScope, NDManager

“Klusters,” “NeuroScope,” and “NDManager” are three integrated modules bundled together for processing and analysis of spike and field potential signals (Hazan et al., 2006). Klusters performs spike sorting using KlustaKwik (see Section 2.3.2) and displays 2D projection of features, spike traces, correlograms, and error matrix view. NeuroScope allows inspection, selection, and event editing of spike signals as well as local field potentials (LFPs). NDManager facilitates experimental and preprocessing parameter management. Available at http://neurosuite.sourceforge.net/.

#### 2.2.4. Brain-system for multivariate AutoRegressive time series (BSMART)

“BSMART” toolbox is written in Matlab/C for spectral analysis of neurophysiological signals (Cui et al., 2008). It provides (multi-)bi-variate AutoRegressive modeling, spectral analysis through coherence and Granger causality, and network analysis. Available at http://www.brain-smart.org/.

#### 2.2.5. Finding information in neural data (FIND)

“FIND” is a platform-independent framework for the analysis of neuronal data based on Matlab (Meier et al., 2008). It provides a unified data import function from various proprietary formats simplifying standardized interfacing with analysis tools and allows analysis of discrete series of spike events, continuous time series, and imaging data. Also, allows simulating multielectrode activity using point-process based stochastic model. Available at http://find.bccn.uni-freiburg.de/.

#### 2.2.6. Spike train analysis toolkit (STAToolkit)

“STAToolkit” is a Matlab/C-hybrid toolbox implementing information theoretic methods to quantify how well the stimuli can be distinguished based on the timing of neuronal firing patterns in a spike train (Goldberg et al., 2009). Available at http://neuroanalysis.org.

#### 2.2.7. PANDORA

“PANDORA” is a Matlab-based toolbox that extracts user-defined characteristics from spike train signals and create numerical database tables from them (Gunay et al., 2009). Further analyses (e.g., drug and parameter effects, spike shape characterization, histogramming and comparison of distributions, cross-correlation, etc.) can then be performed on these tables. Spike detection and feature extraction can also be performed. It is available at http://software.incf.org/software/pandora.

#### 2.2.8. sigTOOL

“sigTOOL” toolbox is written in Matlab and allows direct loading of a wide range of proprietary file formats (Lidierth, 2009). It provides (auto-)cross-correlation, power spectral analysis, and coherence estimation in addition to usual spike train analysis (i.e., ISI, event auto- and cross-correlations, spike-triggered averaging, peri-event time histograms, frequencygrams, etc.). Available at http://sigtool.sourceforge.net/.

#### 2.2.9. Information breakdown ToolBox (ibTB)

“ibTB” is a Matlab-based toolbox which implements information theory methods for spike, LFP, and EEG analysis (Magri et al., 2009). It provides information breakdown technique to decode the encoding of sensory stimuli by different groups of neurons. The source code can be obtained from the publisher's website (http://static-content.springer.com/esm/art%3A10.1186%2F1471-2202-10-81/MediaObjects/1471-2202-10-81-S1.zip).

#### 2.2.10. Chronux

“Chronux” toolbox is developed in Matlab for the analysis of both point process and continuous data (Bokil et al., 2010). It provides spike sorting, and local regression and multitaper spectral analysis of neural signals. Available at http://chronux.org/.

#### 2.2.11. SPKTool

“SPKTool” is coded in Matlab for the detection and analysis of neural spiking activity (Liu et al., 2011). It performs spike detection, feature extraction, manual and semi-automatic clustering of spike trains. Available at http://spktool.sourceforge.net/.

#### 2.2.12. nSTAT

“nSTAT” toolbox is coded in Matlab and performs spike train analysis in time domain (e.g., Kalman Filtering), frequency domain (e.g., multi-taper spectral estimation), and mixed time-frequency domain (e.g., spectrogram) (Cajigas et al., 2012). Available at www.neurostat.mit.edu/nstat/.

#### 2.2.13. SigMate

“SigMate” is a Matlab-based comprehensive framework that allows preprocessing and analysis of EEG, LFPs, and spike signals (Mahmud et al., 2012a). It's main contribution is in the analysis of LFPs which includes data display, file operations, baseline correction, artifact removal, noise characterization, current source density (CSD) analysis, latency estimation from LFPs and CSDs, determination of cortical layer activation order using LFPs and CSDs, and single LFP clustering. The EEG and spike analysis are provided through EEGLAB (see Section 2.1.1) and Wave_Clus (see Section 2.3.1) toolboxes. It can be obtained from https://sites.google.com/site/muftimahmud/codes.

#### 2.2.14. Multivariate granger causality toolbox (MVGC)

“MVGC” is a toolbox written in Matlab that implements WienerGranger causality (G-causality) on multiple equivalent representations of a vector autoregressive model in both time and frequency domains (Barnett and Seth, 2014). It can be applied to neuroelectric, neuromagnetic, and fMRI signals and can be obtained from http://www.sussex.ac.uk/sackler/mvgc/.

#### 2.2.15. QSpike tools

“QSpike Tools” is a Linux/Unix-based cloud-computing framework, modeled using client-server architecture and developed in Matlab / Bash scripts^2^, for processing and analysis of extracellular spike trains (Mahmud et al., 2014). It performs batch preprocessing of CPU-intensive operations for each channel (e.g., filtering, multi-unit activity detection, spike-sorting, etc.), in parallel, by delegating them to a multi-core computer or to a computers cluster. It can be obtained from https://sites.google.com/site/qspiketool/.

### 2.3. Toolboxes for spike sorting

As seen in the literature, majority of the efforts have been devoted in developing tools for spike sorting and analysis. A recent review by Rey et al. outlines the basic concepts of spike sorting, applicability requirements, and shortcoming of currently available algorithms (Rey et al., 2015). Detailing all spike-sorting packages and their functionalities would require a complete review, therefore, here we restrict our discussion to some of the popular open-source toolboxes.

#### 2.3.1. Wave_Clus

“Wave_Clus” is the most popular spike sorting package to date. Developed in Matlab, it uses wavelet transformation based feature selection method and superparamagnetic clustering (Blatt et al., 1996) method to sort the spikes into different classes (Quian Quiroga et al., 2004). It is available at https://vis.caltech.edu/~rodri/Wave_clus/Wave_clus_home.htm.

#### 2.3.2. KlustaKwik

“KlustaKwik” is a stand-alone program written in C++ for automatic clustering analysis (Harris et al., 2000) by fitting a mixture of Gaussians and masked expectation-maximization (Kadir et al., 2014; Rossant et al., 2016). Download link is https://github.com/klusta-team/klustakwik.

#### 2.3.3. OSort

“OSort” is a template-based, unsupervised, online spike sorting algorithm written in Matlab (Rutishauser et al., 2006). It uses residual-sum-of-squares based distance method and custom thresholds to on-the-fly sort the recorded spikes. Available at http://www.urut.ch/new/serendipity/index.php?/pages/osort.html.

#### 2.3.4. SpikeOMatic

“SpikeOMatic” is a spike sorting package developed in R (Pouzat and Chaffiol, 2009). It implements Gaussian Mixture and Dynamic Hidden Markov Models using expectation-maximization and Markov Chain Monte Carlo methods, respectively. Available at http://www.biomedicale.univ-paris5.fr/SpikeOMatic/.

#### 2.3.5. Spyke

“Spyke” is a Python based toolbox for visualizing, navigating, and spike sorting of high-density multichannel extracellular spikes (Spacek et al., 2009). It uses PCA for dimensionality reduction and modified gradient ascent clustering algorithm (Fukunaga and Hostetler, 1975; Swindale and Spacek, 2014) to classify the features. Available at http://spyke.github.io/.

#### 2.3.6. UltraMegaSort2000

“UltraMegaSort2000” is a Matlab based toolbox for spike detection and clustering which implements a hierarchical clustering scheme using similarities of spike shape and spike timing statistics, and provides false-positive and false-negative errors as quality evaluation metrics (Fee et al., 1996; Hill et al., 2011). Available at http://physics.ucsd.edu/neurophysics/software.php.

#### 2.3.7. EToS

“EToS” is a spike sorting toolbox written in C++ implementing multimodality-weighted PCA and variational Bayes for student's *t* mixture model (Takekawa et al., 2012). The spike sorting code is parallelized through OpenMP (www.openmp.org) and available at http://etos.sourceforge.net/.

#### 2.3.8. MClust

“MClust” is a spike sorting toolbox developed in Matlab. It supports both manual and automated clustering with possibility to manual feature selection (Redish, 2014). It can be obtained from http://redishlab.neuroscience.umn.edu/MClust/MClust.html.

#### 2.3.9. NEV2lkit

“NEV2lKit” is a package written in C++ with routines for analysis, visualization and classification of spikes (Bongard et al., 2014). Its results are accurate, efficient and consistency across experiments. Available at http://nev2lkit.sourceforge.net/.

#### 2.3.10. WIToolbox

“WIToolbox” implements a combination of wavelet transform and information theory using Matlab for better classification of spikes on the occasions of spike time-jitter, background noise, and sample size problem (Lopes-dos Santos et al., 2015). Available at www.le.ac.uk/csn/WI.

## 3. Sharing of analysis tools and experimental data

Making available to the community analysis toolboxes for easy and efficient handling of massive neuronal data is just a part of the solution. The other part is the availability of infrastructures which would allow these tools and the experimental data to be shared. Computational neuroscientists are putting constant and significant efforts in building and refining “Neuroinformatics” infrastructures, as outlined below, for making data, tools, and resources electronically accessible over the web (Ascoli, 2006b) which is believed to help and facilitate the standardization, benchmarking process, and foster collaborative research (Mahmud et al., 2012b). As quoted by Prof. Jan G. Bjaalie, “Neuroinformatics applies the methods and approaches required for large scale data integration and thereby paves the way toward understanding the brain”^3^.

### 3.1. Neuroshare

The neuroshare (http://neuroshare.sourceforge.net/) framework started with the goal to create and support open data file format specifications for neurophysiology, a set of open libraries to access those data, and open-source software tools for their analysis. This is particularly important when the community faces a situation where there are many proprietary neuronal signal file formats used by different acquisition softwares. Leveraging the “Neuroshare API,” the framework aims at standardizing the access to individual file formats of neurophysiological experiment data by creating low-level handling and processing tools. However, this has been designed to be achieved in two subsequent phases: (i) creation of open library and format standards for the experimental data, and (ii) developing free and open-source tools for low-level handling and processing of the data. Currently, it provides eight Neuroshare-compliant digital link libraries (DLLs) to access raw data files recorded with proprietary acquisition setups, e.g., Alpha-Omega, Blackrock Microsystems, Cambrige Electronic Design, Multichannel Systems, NeuroExplorer, Plexon, RC Electronics, and Tucker-Davis Technologies.

### 3.2. International neuroinformatics coordinating facility (INCF)

To facilitate tools and data sharing and fostering development in the field of Neuroinformatics, an organization called International Neuroinformatics Coordinating Facility (INCF, www.incf.org) was formed by 12 member countries of the Organization for Economic Co-operation and Development (OECD, www.oecd.org/). Financed by Belgium, Czech Republic, Finland, France, Germany, Italy, Japan, The Netherlands, Norway, Sweden, Switzerland, the United States, and the European Commission, many of these member countries have their own nodes to provide this facility locally (Rautenberg et al., 2011). Quoting from an article by the Executive Director of INCF during 2006–2008, who defined it's aims to be (Bjaalie and Grillner, 2007):

quote

- coordinate and foster international activities in Neuroinformatics;
- contribute to the development of scalable, portable, and extensible applications that can be used for furthering our knowledge of the human brain and its diseases;
- contribute to the development and maintenance of specific database and other computational infrastructures and support mechanisms; and
- focus on developing mechanisms for the seamless flow of information and knowledge between academia, private enterprizes, and the publication industry.

unquote

### 3.3. Code analysis, repository and modeling for e-Neuroscience (CARMEN)

The Code Analysis, Repository and Modeling for e-Neuroscience (CARMEN) project was one of its kind in developing a virtual neuroscience laboratory, specially for electrophysiology data, facilitating e-Neuroscience through creating a unique infrastructure for data and tools sharing and services (Watson et al., 2010). These secure services allow a user to curate data and analysis code to defined storages, document experimental protocols, and execute data analysis (Fletcher et al., 2008). The data as such cannot be curated to the databases of CARMEN without having a proper metadata description about it. This description is essential for accessing correct data out of the thousands of available datasets and interpreting them using the appropriate analysis codes (Jessop et al., 2010).

The CARMEN framework currently supports analysis codes written in Matlab, Python, C/C++, and R. The users may upload their codes, in the form of non-interactive standalone command-line applications, wrapping them using a Service Builder tool to create a suitable service format to be executed on the platform (Weeks et al., 2013).

Recently, a programming document demonstrated the usage of a curated repository of multielectrode array recordings of spontaneous activity from mouse and ferret retina. The mentioned dataset was in HD5^4^ format (a format for hierarchical data organization), and the document outlined the guide to be followed for the efficient usage of the CARMEN software workflow. Moreover, the dataset structure along with examples of reproducible research using those data files were reported (Eglen et al., 2014).

### 3.4. Neurodata without borders: neurophysiology (NWB:N)

To facilitate research reproducibility and to have an opportunity to explore someone else's data, data standardization is a must. The Neurodata Without Borders: Neurophysiology (NWB:N, http://www.nwb.org/) is an initiative aiming at promoting data standardization and sharing. Since it's infancy, the NWB:N has been keen on producing a common data format for recordings and metadata of cellular electrophysiology which has recently been released along with a sample dataset (Teeters et al., 2015).

## 4. Challenges and future perspectives

Secure infrastructures are vital for the success of large-scale, multi-institutional Neuroinformatics research. It is foreseeable that Neuroinformatics research facilities shall be capable of integrating data seamlessly from different sources for data sharing, but also they should be secure enough to address challenging issues like –

- research collaboration with the option to protect their proprietary data,
- user friendliness allowing users with minimal information technology skills to explore, navigate, and use scientific data and services provided by the environment.

In the recent years, the emergence and popularity of distributed computing render an opportunity to share resources that otherwise require more effort. In particular, cloud computing and service oriented architecture open novel avenues necessary to foster collaborative neuronal signal analysis through distributed infrastructure. These approaches allow better representation of responsibilities taken by the different users in accordance to their granted privileges. In our opinion, the development is expected toward:

- Design and implementation of secure and protected systems;
- Advance on cloud based web applications;
- Facilitate easy deployment of data;
- Reusability and sharing of tools with adaptability to changing requirements;
- Empower researchers to share functionalities that they want to publish.

Based on the current state-of-the-art, we identified few challenges that require immediate attention of the community, a few are indicated below:

1. Over the last few years, the neuroscientists have put together quite a few useful neuroimage repositories and their analysis tools (Eickhoff et al., 2016), but neurophysiology is lagging behind. Though there exist a few individual databases (e.g., http://brainliner.jp/, http://www.g-node.org/, https://www.ieeg.org/, etc.), they are very poor in comparison to their imaging counterpart (Tripathy et al., 2014).
2. With the actual acquisition systems and the needed data formats changes, inter-operability and data conversion is still a nightmare due to the lack of widely adopted standards. In addition, when the data are being curated in a databases, the data-description through metadata is again incompatible among different labs/curators which also hampers in conducting meaningful analyses using data from another lab. This unnecessarily increases the time and effort required for data discovery and analysis.
3. Due to the practical problem of rapid and customized analyses, most of the labs develop their own analysis scripts and perform their required analyses. This approach has severe drawbacks on the global scale: interoperability, compatibility, and sharing of tools with other laboratories are highly restricted. Thus, the problem of creating a common set of analyses and the availability of benchmark analysis tools are yet to be addressed.
4. Though the price of computing power has reduced significantly over the years, yet the power required to demystify large neuronal ensembles is still alarmingly high. From a Neuroinformatics perspective, availability of powerful international computing facilities will greatly facilitate remote, automated, and standardized multichannel neuronal signal processing and analysis.
5. Cloud computing's popularity is rapidly growing. Exploiting the bliss of distributed computing, a concept of Competitor-to-Collaborator would be very interesting where small clusters of laboratories working on similar research questions would share their resources and tools through a unified cloud-based framework for the other laboratories to be used as web-services.

## Author contributions

MM performed the reported study. MM wrote and SV edited the paper. Both authors have seen and approved the final manuscript.

### Conflict of interest statement

The authors declare that the research was conducted in the absence of any commercial or financial relationships that could be construed as a potential conflict of interest.

## Footnotes

^1^https://www.perl.org/.

^2^https://en.wikipedia.org/wiki/Bash_(Unix_shell).

^3^https://www.incf.org/community/people/bjaalie/person_view.

^4^https://www.hdfgroup.org/HDF5/.

## References

[B1] Akil H. Martone M. E. Van Essen D. C. (2011). Challenges and opportunities in mining neuroscience data. Science 331, 708–712. 10.1126/science.1199305 21311009 PMC3102049

[B2] Al-Subari K. Al-Baddai S. Tome A. M. Goldhacker M. Faltermeier R. Lang E. W. (2015). EMDLAB: a toolbox for analysis of single-trial EEG dynamics using empirical mode decomposition. J. Neurosci. Methods 253, 193–205. 10.1016/j.jneumeth.2015.06.020 26162614

[B3] Ascoli G. A. (2006a). Mobilizing the base of neuroscience data: the case of neuronal morphologies. Nat. Rev. Neurosci. 7, 318–324. 10.1038/nrn1885 16552417

[B4] Ascoli G. A. (2006b). The ups and downs of neuroscience shares. Neuroinformatics 4, 213–216. 10.1385/NI:4:3:213 16943627

[B5] Baccala L. A. Sameshima K. (2001). Partial directed coherence: a new concept in neural structure determination. Biol. Cybern. 84, 463–474. 10.1007/pl00007990 11417058

[B6] Barnett L. Seth A. K. (2014). The MVGC multivariate Granger causality toolbox: a new approach to Granger-causal inference. J. Neurosci. Methods 223, 50–68. 10.1016/j.jneumeth.2013.10.018 24200508

[B7] Berenyi A. Somogyvari Z. Nagy A. J. Roux L. Long J. D. Fujisawa S.. (2014). Large-scale, high-density (up to 512 channels) recording of local circuits in behaving animals. J. Neurophysiol. 111, 1132–1149. 10.1152/jn.00785.2013 24353300 PMC3949233

[B8] Bigdely-Shamlo N. Mullen T. Kothe C. Su K. M. Robbins K. A. (2015). The PREP pipeline: standardized preprocessing for large-scale EEG analysis. Front. Neuroinform. 9:16. 10.3389/fninf.2015.00016 26150785 PMC4471356

[B9] Billinger M. Brunner C. Muller-Putz G. R. (2014). SCoT: a python toolbox for EEG source connectivity. Front. Neuroinform. 8:22. 10.3389/fninf.2014.00022 24653694 PMC3949292

[B10] Bjaalie J. G. Grillner S. (2007). Global neuroinformatics: the international neuroinformatics coordinating facility. J. Neurosci. 27, 3613–3615. 10.1523/jneurosci.0558-07.2007 17409224 PMC6672406

[B11] Blatt M. Wiseman S. Domany E. (1996). Superparamagnetic clustering of data. Phys. Rev. Lett. 76, 3251–3254. 10.1103/physrevlett.76.3251 10060920

[B12] Bokil H. Andrews P. Kulkarni J. E. Mehta S. Mitra P. P. (2010). Chronux: a platform for analyzing neural signals. J. Neurosci. Methods 192, 146–151. 10.1016/j.jneumeth.2010.06.020 20637804 PMC2934871

[B13] Bongard M. Micol D. Fernandez E. (2014). NEV2lkit: a new open source tool for handling neuronal event files from multi-electrode recordings. Int. J. Neural Syst. 24, 1450009. 10.1142/s0129065714500099 24694167

[B14] Bonomini M. P. Ferrandez J. M. Bolea J. A. Fernandez E. (2005). DATA-MEAns: an open source tool for the classification and management of neural ensemble recordings. J. Neurosci. Methods 148, 137–146. 10.1016/j.jneumeth.2005.04.008 15970333

[B15] Briggman K. L. Denk W. (2006). Towards neural circuit reconstruction with volume electron microscopy techniques. Curr. Opin. Neurobiol. 16, 562–570. 10.1016/j.conb.2006.08.010 16962767

[B16] Brown E. N. Kass R. E. Mitra P. P. (2004). Multiple neural spike train data analysis: state-of-the-art and future challenges. Nat. Neurosci. 7, 456–461. 10.1038/nn1228 15114358

[B17] Buzsaki G. (2004). Large-scale recording of neuronal ensembles. Nat. Neurosci. 7, 446–451. 10.1038/nn1233 15114356

[B18] Buzsaki G. Anastassiou C. A. Koch C. (2012). The origin of extracellular fields and currents–EEG, ECoG, LFP and spikes. Nat. Rev. Neurosci. 13, 407–420. 10.1038/nrn3241 22595786 PMC4907333

[B19] Cajigas I. Malik W. Q. Brown E. N. (2012). nSTAT: open-source neural spike train analysis toolbox for matlab. J. Neurosci. Methods 211, 245–264. 10.1016/j.jneumeth.2012.08.009 22981419 PMC3491120

[B20] Celeux G. Govaert G. (1992). A classification em algorithm for clustering and two stochastic versions. Comput. Stat. Data Anal., 14, 315–332. 10.1016/0167-9473(92)90042-e

[B21] Chao A. Shen T.-J. (2003). Nonparametric estimation of shannon's index of diversity when there are unseen species in sample. Environ. Ecol. Stat. 10, 429–443. 10.1023/A:1026096204727

[B22] Cleveland W. S. (1979). Robust locally weighted regression and smoothing scatterplots. J. Am. Stat. Assoc. 74, 829–836. 10.1080/01621459.1979.10481038

[B23] Comon P. (1994). Higher order statistics independent component analysis, a new concept? Signal Process. 36, 287–314.

[B24] Cover T. Hart P. (1967). Nearest neighbor pattern classification. IEEE Trans. Inform. Theory 13, 21–27. 10.1109/tit.1967.1053964

[B25] Cui J. Xu L. Bressler S. L. Ding M. Liang H. (2008). BSMART: a matlab/c toolbox for analysis of multichannel neural time series. Neural Netw. 21, 1094–1104. 10.1016/j.neunet.2008.05.007 18599267 PMC2585694

[B26] Dale A. M. Sereno M. I. (1993). Improved localization of cortical activity by combining eeg and meg with mri cortical surface reconstruction - a linear-approach. J. Cogn. Neurosci. 5, 162–176. 23972151 10.1162/jocn.1993.5.2.162

[B27] de Cheveigné A. Parra L. C. (2014). Joint decorrelation, a versatile tool for multichannel data analysis. Neuroimage 98, 487–505. 10.1016/j.neuroimage.2014.05.068 24990357

[B28] De Schutter E. (2010). Data publishing and scientific journals: the future of the scientific paper in a world of shared data. Neuroinformatics 8, 151–153. 10.1007/s12021-010-9084-8 20835853

[B29] Delorme A. Makeig S. (2004). EEGLAB: an open source toolbox for analysis of single-trial eeg dynamics including independent component analysis. J. Neurosci. Methods 134, 9–21. 10.1016/j.jneumeth.2003.10.009 15102499

[B30] Duda R. O. Hart P. E. Stork D. G. (2000). Pattern Classification, 2nd Edn. New York, NY: Wiley-Interscience.

[B31] Eglen S. J. Weeks M. Jessop M. Simonotto J. Jackson T. Sernagor E. (2014). A data repository and analysis framework for spontaneous neural activity recordings in developing retina. Gigascience 3, 3. 10.1186/2047-217X-3-3 24666584 PMC4076503

[B32] Eickhoff S. Nichols T. E. Van Horn J. D. Turner J. A. (2016). Sharing the wealth: neuroimaging data repositories. Neuroimage 124(Pt B), 1065–1068. 10.1016/j.neuroimage.2015.10.079 26574120 PMC5463741

[B33] Einevoll G. T. Franke F. Hagen E. Pouzat C. Harris K. D. (2012). Towards reliable spike-train recordings from thousands of neurons with multielectrodes. Curr. Opin. Neurobiol. 22, 11–17. 10.1016/j.conb.2011.10.001 22023727 PMC3314330

[B34] Fee M. S. Mitra P. P. Kleinfeld D. (1996). Automatic sorting of multiple unit neuronal signals in the presence of anisotropic and non-gaussian variability. J. Neurosci. Methods 69, 175–188. 10.1016/s0165-0270(96)00050-7 8946321

[B35] Fletcher M. Liang B. Smith L. Knowles A. Jackson T. Jessop M. (2008). Neural network based pattern matching and spike detection tools and services–in the CARMEN neuroinformatics project. Neural Netw. 21, 1076–1084. 10.1016/j.neunet.2008.06.009 18674883

[B36] Freeman J. (2015). Open source tools for large-scale neuroscience. Curr. Opin. Neurobiol. 32, 156–163. 10.1016/j.conb.2015.04.002 25982977

[B37] Friston K. J. Bastos A. M. Pinotsis D. Litvak V. (2015). LFP and oscillations–what do they tell us? Curr. Opin. Neurobiol. 31, 1–6. 10.1016/j.conb.2014.05.004 25079053 PMC4376394

[B38] Fukunaga K. Hostetler L. (1975). The estimation of the gradient of a density function, with applications in pattern recognition. IEEE Trans. Inform. Theory 21, 32–40. 10.1109/tit.1975.1055330

[B39] Gardner D. Akil H. Ascoli G. A. Bowden D. M. Bug W. Donohue D. E.. (2008). The neuroscience information framework: a data and knowledge environment for neuroscience. Neuroinformatics 6, 149–160. 10.1007/s12021-008-9024-z 18946742 PMC2661130

[B40] Giocomo L. M. (2015). Large scale *in vivo* recordings to study neuronal biophysics. Curr. Opin. Neurobiol. 32, 1–7. 10.1016/j.conb.2014.09.009 25291296

[B41] Goldberg D. Victor J. Gardner E. Gardner D. (2009). Spike train analysis toolkit: enabling wider application of information–theoretic techniques to neurophysiology. Neuroinformatics 7, 165–178. 10.1007/s12021-009-9049-y 19475519 PMC2818590

[B42] Granger C. W. J. (1969). Investigating causal relations by econometric models and cross-spectral methods. Econometrica 37, 424–438.

[B43] Grech R. Cassar T. Muscat J. Camilleri K. P. Fabri S. G. Zervakis M.. (2008). Review on solving the inverse problem in eeg source analysis. J. Neuroeng. Rehabil. 5, 25. 10.1186/1743-0003-5-25 18990257 PMC2605581

[B44] Gunay C. Edgerton J. Li S. Sangrey T. Prinz A. A. Jaeger D. (2009). Database analysis of simulated and recorded electrophysiological datasets with pandora's toolbox. Neuroinformatics 7, 93–111. 10.1007/s12021-009-9048-z 19475520 PMC2786174

[B45] Hallez H. Vanrumste B. Grech R. Muscat J. De Clercq W. Vergult A.. (2007). Review on solving the forward problem in EEG source analysis. J. Neuroeng. Rehabil. 4, 46. 10.1186/1743-0003-4-46 18053144 PMC2234413

[B46] Hanke M. Halchenko Y. O. Sederberg P. B. Olivetti E. Frund I. Rieger J. W.. (2009). PyMVPA: a unifying approach to the analysis of neuroscientific data. Front. Neuroinform. 3:3. 10.3389/neuro.11.003.2009 19212459 PMC2638552

[B47] Harris K. D. Henze D. A. Csicsvari J. Hirase H. Buzsaki G. (2000). Accuracy of tetrode spike separation as determined by simultaneous intracellular and extracellular measurements. J. Neurophysiol. 84, 401–414. Avaliable online at: http://jn.physiology.org/content/84/1/401.long 10899214 10.1152/jn.2000.84.1.401

[B48] Harshman R. A. (1970). Foundations of the PARAFAC procedure: models and conditions for an ëxplanatorym¨ulti-modal factor analysis, in UCLA Working Papers in Phonetics (Los Angeles, CA), Vol. 16, 84.

[B49] Hazan L. Zugaro M. Buzsaki G. (2006). Klusters, NeuroScope, NDManager: a free software suite for neurophysiological data processing and visualization. J. Neurosci. Methods 155, 207–216. 10.1016/j.jneumeth.2006.01.017 16580733

[B50] He B. Dai Y. Astolfi L. Babiloni F. Yuan H. Yang L. (2011). eConnectome: a Matlab toolbox for mapping and imaging of brain functional connectivity. J. Neurosci. Methods 195, 261–269. 10.1016/j.jneumeth.2010.11.015 21130115 PMC3244474

[B51] Hill D. N. Mehta S. B. Kleinfeld D. (2011). Quality metrics to accompany spike sorting of extracellular signals. J. Neurosci. 31, 8699–8705. 10.1523/JNEUROSCI.0971-11.2011 21677152 PMC3123734

[B52] Ince R. A. Mazzoni A. Petersen R. S. Panzeri S. (2010). Open source tools for the information theoretic analysis of neural data. Front. Neurosci. 4:62. 10.3389/neuro.01.011.2010 20730105 PMC2891486

[B53] Jessop M. Weeks M. Austin J. (2010). CARMEN: a practical approach to metadata management. Philos. Trans. A Math. Phys. Eng. Sci. 368, 4147–4159. 10.1098/rsta.2010.0147 20679128

[B54] Johnson D. H. Sinanovic S. (2001). Symmetrizing the Kullback-Leibler Distance. Available online at: http://www.ece.rice.edu/~dhj/resistor.pdf (Accessed: April 15, 2016).

[B55] Kadir S. N. Goodman D. F. Harris K. D. (2014). High-dimensional cluster analysis with the masked em algorithm. Neural Comput. 26, 2379–2394. 10.1162/NECO_a_00661 25149694 PMC4298163

[B56] Kaminski M. J. Blinowska K. J. (1991). A new method of the description of the information flow in the brain structures. Biol. Cybern. 65, 203–210. 10.1007/BF00198091 1912013

[B57] Khodagholy D. Gelinas J. N. Thesen T. Doyle W. Devinsky O. Malliaras G. G.. (2015). NeuroGrid: recording action potentials from the surface of the brain. Nat. Neurosci. 18, 310–315. 10.1038/nn.3905 25531570 PMC4308485

[B58] Koles Z. J. Lazar M. S. Zhou S. Z. (1990). Spatial patterns underlying population differences in the background EEG. Brain Topogr. 2, 275–284. 10.1007/BF01129656 2223384

[B59] Krishnapuram B. Carin L. Figueiredo M. A. Hartemink A. J. (2005). Sparse multinomial logistic regression: fast algorithms and generalization bounds. IEEE Trans. Pattern Anal. Mach. Intell. 27, 957–968. 10.1109/TPAMI.2005.127 15943426

[B60] Kullback S. Leibler R. A. (1951). On information and sufficiency. Ann. Math. Stat. 22, 79–86. 10.1214/aoms/1177729694

[B61] Lenkov D. N. Volnova A. B. Pope A. R. Tsytsarev V. (2013). Advantages and limitations of brain imaging methods in the research of absence epilepsy in humans and animal models. J. Neurosci. Methods 212, 195–202. 10.1016/j.jneumeth.2012.10.018 23137652

[B62] Levinson N. (1946). The wiener (root mean square) error criterion in filter design and prediction. J. Math. Phys. 25, 261–278. 10.1002/sapm1946251261

[B63] Lidierth M. (2009). sigTOOL: a Matlab-based environment for sharing laboratory-developed software to analyze biological signals. J. Neurosci. Methods 178, 188–196. 10.1016/j.jneumeth.2008.11.004 19056423

[B64] Liu X. Q. Wu X. Liu C. (2011). SPKtool: an open source toolbox for electrophysiological data processing, in 2011 International Conference on Biomedical Engineering and Informatics (BMEI) (Shanghai: IEEE), Vol. 2, 854–857. 10.1109/BMEI.2011.6098451

[B65] Loader C. (1999). Local Regression and Likelihood. New York, NY: Springer-Verlag.

[B66] Lopes-dos Santos V. Panzeri S. Kayser C. Diamond M. E. Quian Quiroga R. (2015). Extracting information in spike time patterns with wavelets and information theory. J. Neurophysiol. 113, 1015–1033. 10.1152/jn.00380.2014 25392163 PMC4312869

[B67] Ma S. K. (1981). Calculation of entropy from data of motion. J. Stat. Phys. 26, 221–240. 10.1007/BF01013169

[B68] MacQueen J. (1967). Some methods for classification and analysis of multivariate observations, in Proceedings of the 5th Berkeley Symposium on Mathematical Statistics and Probability (Berkeley, CA), 1965/66 Vol. 1, 281–297.

[B69] Magri C. Whittingstall K. Singh V. Logothetis N. Panzeri S. (2009). A toolbox for the fast information analysis of multiple-site lfp, eeg and spike train recordings. BMC Neurosci. 10:81. 10.1186/1471-2202-10-81 19607698 PMC2723115

[B70] Mahmud M. Bertoldo A. Girardi S. Maschietto M. Vassanelli S. (2010a). SigMate: a Matlab-based neuronal signal processing tool. Conf. Proc. IEEE Eng. Med. Biol. Soc. 2010, 1352–1355. 10.1109/iembs.2010.5626747 21096329

[B71] Mahmud M. Bertoldo A. Girardi S. Maschietto M. Vassanelli S. (2012a). SigMate: a Matlab-based automated tool for extracellular neuronal signal processing and analysis. J. Neurosci. Methods 207, 97–112. 10.1016/j.jneumeth.2012.03.009 22513383

[B72] Mahmud M. Bertoldo A. Maschietto M. Girardi S. Vassanelli S. (2010b). Automatic detection of layer activation order in information processing pathways of rat barrel cortex under mechanical whisker stimulation. Conf. Proc. IEEE Eng. Med. Biol. Soc. 2010, 6095–6098. 10.1109/iembs.2010.5627639 21097132

[B73] Mahmud M. Cecchetto C. Vassanelli S. (2016). An automated method for characterization of evoked single-trial local field potentials recorded from rat barrel cortex under mechanical whisker stimulation. Cogn. Comput. 1–11. 10.1007/s12559-016-9399-3. [Epub ahead of print].

[B74] Mahmud M. Pasqualotto E. Bertoldo A. Girardi S. Maschietto M. Vassanelli S. (2011). An automated method for detection of layer activation order in information processing pathway of rat barrel cortex under mechanical whisker stimulation. J. Neurosci. Methods 196, 141–150. 10.1016/j.jneumeth.2010.11.024 21145917

[B75] Mahmud M. Pulizzi R. Vasilaki E. Giugliano M. (2014). QSpike tools: a generic framework for parallel batch preprocessing of extracellular neuronal signals recorded by substrate microelectrode arrays. Front. Neuroinform. 8:26. 10.3389/fninf.2014.00026 24678297 PMC3958706

[B76] Mahmud M. Rahman M. M. Travalin D. Raif P. Hussain A. (2012b). Service oriented architecture based web application model for collaborative biomedical signal analysis. Biomed. Tech. 57(Suppl. 1), 780–783. 10.1515/bmt-2012-4412 23096278

[B77] Mahmud M. Travalin D. Bertoldo A. Girardi S. Maschietto M. Vassanelli S. (2012c). An automated classification method for single sweep local field potentials recorded from rat barrel cortex under mechanical whisker stimulation. J. Med. Biol. Eng. 32, 397–404. 10.5405/jmbe.923

[B78] Marathe A. R. Ries A. J. McDowell K. (2014). Sliding HDCA: single-trial EEG classification to overcome and quantify temporal variability. IEEE Trans. Neural Syst. Rehabil. Eng. 22, 201–211. 10.1109/TNSRE.2014.2304884 24608681

[B79] Meier R. Egert U. Aertsen A. Nawrot M. P. (2008). FIND – a unified framework for neural data analysis. Neural Netw. 21, 1085–1093. 10.1016/j.neunet.2008.06.019 18692360

[B80] Mikula S. Binding J. Denk W. (2012). Staining and embedding the whole mouse brain for electron microscopy. Nat. Methods 9, 1198–1201. 10.1038/nmeth.2213 23085613

[B81] Misra N. Singh H. Demchuk E. (2005). Estimation of the entropy of a multivariate normal distribution. J. Multivariate Anal. 92, 324–342. 10.1016/j.jmva.2003.10.003

[B82] Mitra P. Pesaran B. (1999). Analysis of dynamic brain imaging data. Biophys. J. 76, 691–708. 10.1016/s0006-3495(99)77236-x 9929474 PMC1300074

[B83] Montemurro M. A. Senatore R. Panzeri S. (2007). Tight data-robust bounds to mutual information combining shuffling and model selection techniques. Neural Comput. 19, 2913–2957. 10.1162/neco.2007.19.11.2913 17883346

[B84] Morup M. Hansen L. K. Arnfred S. M. (2007). ERPWAVELAB: a toolbox for multi-channel analysis of time-frequency transformed event related potentials. J. Neurosci. Methods 161, 361–368. 10.1016/j.jneumeth.2006.11.008 17204335

[B85] Mukhopadhyay S. Ray G. C. (1998). A new interpretation of nonlinear energy operator and its efficacy in spike detection. IEEE Trans. Biomed. Eng. 45, 180–187. 10.1109/10.661266 9473841

[B86] Nawrot P. M. Aertsen A. Rotter S. (2003). Elimination of response latency variability in neuronal spike trains. Biol. Cybern. 88, 321–334. 10.1007/s00422-002-0391-5 12750895

[B87] Nicolelis M. A. Lebedev M. A. (2009). Principles of neural ensemble physiology underlying the operation of brain-machine interfaces. Nat. Rev. Neurosci. 10, 530–540. 10.1038/nrn2653 19543222

[B88] Oostenveld R. Fries P. Maris E. Schoffelen J. M. (2011). FieldTrip: open source software for advanced analysis of MEG, EEG, and invasive electrophysiological data. Comput. Intell. Neurosci. 2011, 156869. 10.1155/2011/156869 21253357 PMC3021840

[B89] Optican L. M. Gawne T. J. Richmond B. J. Joseph P. J. (1991). Unbiased measures of transmitted information and channel capacity from multivariate neuronal data. Biol. Cybern. 65, 305–310. 10.1007/bf00216963 1742368

[B90] Paninski L. (2003). Estimation of entropy and mutual information. Neural Comput. 15, 1191–1253. 10.1162/089976603321780272

[B91] Paninski L. Pillow J. Lewi J. (2007). Statistical models for neural encoding, decoding, and optimal stimulus design. Prog. Brain Res. 165, 493–507. 10.1016/s0079-6123(06)65031-0 17925266

[B92] Panzeri S. Treves, A. (1996). Analytical estimates of limited sampling biases in different information measures. Netw. Comput. Neural Syst. 7, 87–107. 10.1088/0954-898X/7/1/006 29480146

[B93] Pascual-Marqui R. D. Esslen M. Kochi K. Lehmann D. (2002). Functional imaging with low-resolution brain electromagnetic tomography (loreta): a review. Methods Find Exp. Clin. Pharmacol. 24(Suppl. C), 91–95. 12575492

[B94] Percival D. B. Walden A. T. (1993). Multitaper spectral estimation, in Spectral Analysis for Physical Applications: Multitaper and Conventional Univariate Techniques (New York, NY: Cambridge University Press), 331–377. 10.1017/cbo9780511622762.010

[B95] Perrier V. Philipovitch T. Basdevant C. (1995). Wavelet spectra compared to fourier spectra. J. Math. Phys. 36, 1506–1519. 10.1063/1.531340

[B96] Pouzat C. Chaffiol A. (2009). Automatic spike train analysis and report generation. an implementation with r, r2html and star. J. Neurosci. Methods 181, 119–144. 10.1016/j.jneumeth.2009.01.037 19473708

[B97] Quian Quiroga R. Kreuz T. Grassberger P. (2002). Event synchronization: a simple and fast method to measure synchronicity and time delay patterns. Phys. Rev. E 66:041904. 10.1103/physreve.66.041904 12443232

[B98] Quian Quiroga R. Nadasdy Z. Ben-Shaul Y. (2004). Unsupervised spike detection and sorting with wavelets and superparamagnetic clustering. Neural Comput. 16, 1661–1687. 10.1162/089976604774201631 15228749

[B99] Rasmussen C. E. Williams C. K. I. (2006). Gaussian Processes for Machine Learning. Adaptive Computation And Machine Learning. Cambridge, MA: MIT Press.

[B100] Rautenberg P. L. Sobolev A. Herz A. V. Wachtler T. (2011). A database system for electrophysiological data, in Transactions on Large-Scale Data- and Knowledge-Centered Systems IV, Volume 6990 of *Lecture Notes in Computer Science*, eds Hameurlain A. Küng J. Wagner R. Böhm C. Eder J. Plant C. (Berlin; Heidelberg: Springer), 1–14. 10.1007/978-3-642-23740-9_1

[B101] Redish A. D. (2014). MClust free-ware spike sorting. Available online at: http://redishlab.neuroscience.umn.edu/MClust/MClust.html (Accessed 07/01/2016).

[B102] Rehman N. Mandic D. P. (2010). Multivariate empirical mode decomposition. Proc. R. Soc. A 466, 1291–1302. 10.1098/rspa.2009.0502

[B103] Rey H. G. Pedreira C. Quian Quiroga R. (2015). Past, present and future of spike sorting techniques. Brain Res. Bull. 119(Pt B), 106–117. 10.1016/j.brainresbull.2015.04.007 25931392 PMC4674014

[B104] Robbins K. A. (2012). EEGVIS: a Matlab toolbox for browsing, exploring, and viewing large datasets. Front. Neuroinform. 6:17. 10.3389/fninf.2012.00017 22654753 PMC3361060

[B105] Rossant C. Kadir S. N. Goodman D. F. Schulman J. Hunter M. L. Saleem A. B.. (2016). Spike sorting for large, dense electrode arrays. Nat. Neurosci. 19, 634–641. 10.1038/nn.4268 26974951 PMC4817237

[B106] Rutishauser U. Schuman E. M. Mamelak A. N. (2006). Online detection and sorting of extracellularly recorded action potentials in human medial temporal lobe recordings, *in vivo*. J. Neurosci. Methods 154, 204–224. 10.1016/j.jneumeth.2005.12.033 16488479

[B107] Rutkowski T. M. Mandic D. P. Cichocki A. Przybyszewski A. W. (2010). EMD approach to multichannel eeg data – the amplitude and phase components clustering analysis. J. Circuit. Syst. Comp. 19, 215–229. 10.1142/s0218126610006037

[B108] Schmidt R. O. (1986). Multiple emitter location and signal parameter-estimation. IEEE Trans. Antennas. Propagat. 34, 276–280. 10.1109/TAP.1986.1143830

[B109] Schofield P. N. Bubela T. Weaver T. Portilla L. Brown S. D. Hancock J. M.. (2009). Post-publication sharing of data and tools. Nature 461, 171–173. 10.1038/461171a 19741686 PMC6711854

[B110] Schröder S. Cecchetto C. Keil S. Mahmud M. Brose E. Dogan O. (2015). CMOS-compatible purely capacitive interfaces for high-density *in-vivo* recording from neural tissue, in Biomedical Circuits and Systems Conference (BioCAS), 2015 (Atlanta, GA: IEEE), 1–4. 10.1109/biocas.2015.7348358

[B111] Sejnowski T. J. Churchland P. S. Movshon J. A. (2014). Putting big data to good use in neuroscience. Nat. Neurosci. 17, 1440–1441. 10.1038/nn.3839 25349909 PMC4224030

[B112] Spacek M. Blanche T. Swindale N. (2009). Python for large-scale electrophysiology. Front. Neuroinform. 2:9. 10.3389/neuro.11.009.2008 19198646 PMC2634532

[B113] Spira M. E. Hai A. (2013). Multi-electrode array technologies for neuroscience and cardiology. Nat. Nanotechnol. 8, 83–94. 10.1038/nnano.2012.265 23380931

[B114] Stam C. J. (2005). Nonlinear dynamical analysis of eeg and meg: review of an emerging field. Clin. Neurophysiol. 116, 2266–2301. 10.1016/j.clinph.2005.06.011 16115797

[B115] Stevenson I. H. Kording K. P. (2011). How advances in neural recording affect data analysis. Nat. Neurosci. 14, 139–142. 10.1038/nn.2731 21270781 PMC3410539

[B116] Strong S. P. Koberle R. de Ruyter van Steveninck R. R. Bialek W. (1998). Entropy and information in neural spike trains. Phys. Rev. Lett. 80, 197–200. 10.1103/physrevlett.80.197

[B117] Swindale N. V. Spacek M. A. (2014). Spike sorting for polytrodes: a divide and conquer approach. Front. Syst. Neurosci. 8:6. 10.3389/fnsys.2014.00006 24574979 PMC3918743

[B118] Takekawa T. Isomura Y. Fukai T. (2012). Spike sorting of heterogeneous neuron types by multimodality-weighted pca and explicit robust variational bayes. Front. Neuroinform. 6:5. 10.3389/fninf.2012.00005 22448159 PMC3307250

[B119] Teeters J. L. Godfrey K. Young R. Dang C. Friedsam C. Wark B.. (2015). Neurodata without borders: creating a common data format for neurophysiology. Neuron 88, 629–634. 10.1016/j.neuron.2015.10.025 26590340

[B120] Thomson D. Chave A. (1991). Jackknifed error estimates for spectra, coherences, and transfer functions, in Advances in Spectrum Analysis and Array Processing (Englewood Cliffs, NJ: Prentice-Hall, Inc.), 58–113.

[B121] Treves A. Panzeri S. (1995). The upward bias in measures of information derived from limited data samples. Neural. Comput. 7, 399–407. 10.1162/neco.1995.7.2.399

[B122] Tripathy S. J. Savitskaya J. Burton S. D. Urban N. N. Gerkin R. C. (2014). Neuroelectro: a window to the world's neuron electrophysiology data. Front. Neuroinform. 8:40. 10.3389/fninf.2014.00040 24808858 PMC4010726

[B123] Tsytsarev V. Taketani M. Schottler F. Tanaka S. Hara M. (2006). A new planar multielectrode array: recording from a rat auditory cortex. J. Neural Eng. 3, 293–298. 10.1088/1741-2560/3/4/006 17124333

[B124] Tucker L. R. (1966). Some mathematical notes on three-mode factor analysis. Psychometrika 31, 279–311. 10.1007/BF02289464 5221127

[B125] Van Horn J. D. Ball C. A. (2008). Domain-specific data sharing in neuroscience: what do we have to learn from each other? Neuroinformatics 6, 117–121. 10.1007/s12021-008-9019-9 18473189 PMC3319052

[B126] Vapnik V. N. (1995). The Nature of Statistical Learning Theory. New York: NY: Springer 10.1007/978-1-4757-2440-0

[B127] Vassanelli S. (2011). Brain-chip interfaces: the present and the future. Proc. Comput. Sci. 7, 61–64. 10.1016/j.procs.2011.12.020

[B128] Vassanelli S. (2014). Multielectrode and multitransistor arrays for *in vivo* recording, in Nanotechnology and Neuroscience: Nano-electronic, Photonic and Mechanical Neuronal Interfacing, eds De Vittorio M. Martiradonna L. Assad J. (New York, NY: Springer), 239–267. 10.1007/978-1-4899-8038-0_8

[B129] Vassanelli S. Mahmud M. Girardi S. Maschietto M. (2012). On the way to large-scale and high-resolution brain-chip interfacing. Cogn. Comput. 4, 71–81. 10.1007/s12559-011-9121-4

[B130] Veen B. D. V. Buckley K. M. (1988). Beamforming: a versatile approach to spatial filtering. IEEE ASSP Magazine 5, 4–24. 10.1109/53.665

[B131] Victor J. D. (2002). Binless strategies for estimation of information from neural data. Phys. Rev. E 66:051903. 10.1103/physreve.66.051903 12513519

[B132] Victor J. D. Purpura K. P. (1997). Metric-space analysis of spike trains: theory, algorithms and application. Netw. Comput. Neural Syst. 8, 127–164. 10.1088/0954-898X_8_2_003

[B133] Wagenaar D. DeMarse T. B. Potter S. M. (2005). MeaBench: a toolset for multi-electrode data acquisition and on-line analysis, in Proceedings of 2nd International IEEE EMBS Conference on Neural Engineering (Arlington, VA), v–viii. 10.1109/cne.2005.1419673

[B134] Watson P. Hiden H. Woodman S. (2010). e-Science Central for CARMEN: science as a service. Concurr. Comput. 22, 2369–2380. 10.1002/cpe.1611

[B135] Weeks M. Jessop M. Fletcher M. Hodge V. Jackson T. Austin J. (2013). The CARMEN software as a service infrastructure. Philos. Trans. A Math. Phys. Eng. Sci. 371:20120080. 10.1098/rsta.2012.0080 23230159

[B136] Wilke C. Ding L. He B. (2008). Estimation of time-varying connectivity patterns through the use of an adaptive directed transfer function. IEEE Trans. Biomed. Eng. 55, 2557–2564. 10.1109/tbme.2008.919885 18990625 PMC2597483

[B137] Wolpert D. H. Wolf D. R. (1995). Estimating functions of probability distributions from a finite set of samples. Phys. Rev. E 52, 6841–6854. 10.1103/physreve.52.6841 9964199

[B138] Zeiler A. Faltermeier R. Tomé A. M. Puntonet C. Brawanski A. Lang E. W. (2012). Weighted sliding empirical mode decomposition for online analysis of biomedical time series. Neural Process. Lett. 37, 21–32. 10.1007/s11063-012-9270-9

